# Effects of Dietary Nitrate Supplementation on High-Intensity Cycling Sprint Performance in Recreationally Active Adults: A Systematic Review and Meta-Analysis

**DOI:** 10.3390/nu16162764

**Published:** 2024-08-19

**Authors:** Rachel Tan, Jordan K. Cass, Isabella G. Lincoln, Lauren E. Wideen, Madelyn J. Nicholl, Trevor J. Molnar, Lewis A. Gough, Stephen J. Bailey, Adam Pennell

**Affiliations:** 1 Natural Sciences Division, Pepperdine University, Malibu, CA 90263, USA; jordy.cass@pepperdine.edu (J.K.C.);; 2 Human Performance and Health Laboratory, Centre for Life and Sport Sciences (CLaSS), Birmingham City University, Birmingham B5 5JU, UK; lewis.gough@bcu.ac.uk; 3 School of Sport, Exercise and Health Sciences, Loughborough University, Loughborough LE11 3TU, UK; s.bailey2@lboro.ac.uk

**Keywords:** nitric oxide, beetroot, ergogenic aid, power

## Abstract

This systematic review and meta-analysis investigated the influence of dietary nitrate supplementation on performance metrics during cycling sprint exercise according to the PRISMA guidelines. Searches were conducted on MEDLINE, PubMed, ScienceDirect, Scopus, and SPORTDiscus databases up to September 2023. Inclusion criteria were healthy recreationally active men and women who consumed nitrate-rich and nitrate-deficient beetroot juice to assess performance outcomes of mean power, peak power, time-to-peak power, and minimum power during 30-s cycling sprints. Risk of bias was assessed using the Cochrane Risk of Bias 2 and TESTEX tools and funnel plots. A random effects model was performed on six studies and showed that dietary nitrate had significant effects on time-to-peak power (SMD: −0.66, 95% CI: −1.127 to −0.192, *p* = 0.006) but not on mean power, peak power, or minimum power. Subgroup analysis revealed that an acute low nitrate dose improved time-to-peak power (SMD: −0.977, 95% CI: −1.524 to −0.430, *p* < 0.001) but not after a multiday moderate nitrate dose (SMD: −0.177, 95% CI: −0.619 to −0.264, *p* = 0.431). These data suggest that acute nitrate supplementation can benefit time-to-peak power during 30-s cycling sprints, but due to the limited availability of data and heterogeneity in methodology, these results should be interpreted with caution. There was insufficient data on women to analyze sex-based differences. Future studies are required to provide insight on how supplementation regimen and population impact the effects of dietary nitrate for enhancing cycling sprint performance.

## 1. Introduction

The ability to produce power is critical for cycling performance outcomes given that the distribution of work, i.e., pacing strategy, is an important consideration that is dependent on the type of event [1,2]. For example, an explosive “all-out” pacing strategy is advantageous for short-duration sprint events, but in contrast, acceleration and power generation in the final “end sprint” during middle-distance events or at the end of a tour stage during long-distance events may be more beneficial [3]. Moreover, external factors (e.g., environmental conditions, race dynamics, hill climbs, etc.) may require a cyclist to adopt variable pacing strategies whereby power output is continually altered on demand to combat various race conditions [4]. Accordingly, strategies to improve cycling power output are of great interest to recreational and competitive cyclists to enhance cycling performance [5]. 

High-intensity interval exercise (i.e., sprint interval training) has been employed by athletes for decades given that this type of training elicits robust physiological [6] and performance adaptations [7,8]. High-intensity interval exercise includes single or repeated intermittent bouts of high-intensity exercise, typically performed above the heavy-intensity domain and up to maximal “all-out” efforts, interspersed by periods of active or passive recovery [9]. One form of interval exercise is the traditional “Wingate” model consisting of 30-s “all-out” maximal efforts set to 7.5% body mass, interspersed by 4 min of recovery and performed on mechanically-braked cycle ergometers [10,11]. To date, Wingate tests and modified Wingate tests are commonly used to assess anaerobic performance in sports [12], with Wingate test peak power [13] and mean power [7] reported to increase following training. Additionally, anaerobic cycling performance from Wingate tests [14,15,16,17,18] and modified Wingate tests [19] have been correlated with improved exercise performance, further highlighting the benefit of including sprint intervals in training programs for sporting performance. While sprint interval exercise can improve cycling performance outcomes, combining ergogenic aids with interval exercise may work synergistically to provide additional (or negative) benefits to either performance or adaptation compared to either intervention alone, which is important to understand for recreational and competitive individuals who seek to improve performance. 

Dietary nitrate supplementation, often administered as concentrated nitrate-rich beetroot juice, has been reported to induce small improvements to endurance cycling exercise performance such as time-to-exhaustion trials, with reported ergogenic effects primarily in recreationally active individuals but not elite-level athletes [20]. These performance effects may be due to improved energetic cost of contraction via modulated calcium-handling processes [21,22,23], improved distribution of blood flow [24], and preferential effects on type II muscle fibers [21,24,25]. Recent advances suggest the possibility for S-nitrosothiols to have a role in modulating calcium handling [26], although further evidence is required [27,28]. Together, in theory, these mechanisms would increase the likelihood of nitrate eliciting ergogenic effects during explosive, high-intensity type exercise. In support of this, a recent meta-analysis reported that dietary nitrate supplementation had small positive effects on high-intensity exercise performance (e.g., improved time-to-peak power, mean power, and total distance covered), but importantly, numerous exercise modalities were included such as cycling, running, and kayaking [29]. Additionally, since cycling sprint performance may be modulated by a myriad of variables (i.e., intensity, work-to-rest ratio, number of sprints, etc. [6]), and that the efficacy of dietary nitrate on power output is influenced by various factors (e.g., supplementation regimen [30]), there is currently a limited understanding on the ergogenicity of dietary nitrate on high-intensity cycling performance. The 30-s Wingate test model and modified Wingate (i.e., non-Wingate) models are widely used in training programs for various sports and as a research model, and correlate well with various aspects of high-intensity exercise performance [19,31,32]. Thus, since dietary nitrate appears to be most efficacious in recreationally active individuals, understanding how dietary nitrate could alter performance in 30-s cycling sprints in recreationally active individuals may have implications for high-intensity exercise in general and exercise performance in other exercise modalities. Therefore, the purpose of this systematic review and meta-analysis was to examine the efficacy of dietary nitrate supplementation on power output metrics during 30-s high-intensity cycling sprints in healthy recreationally active adults.

## 2. Materials and Methods

The protocol for the present systematic review and meta-analysis was registered on the Open Science Framework (OSF) database (osf.io/7kjax) on 13 June 2024 and is reported according to the Preferred Reporting Items for Systematic Reviews and Meta-Analysis (PRISMA) guidelines [33] and the PICOS (participants, interventions, comparators, outcomes, and study design) criteria [34] (Table 1).

### 2.1. Search Strategy and Study Selection

The literature search was conducted on MEDLINE, PubMed, ScienceDirect, Scopus, and SPORTDiscus databases and included all literature published before 14 September 2023. A combination of keywords and subject headings were used as search terms: (“nitrate” OR “beetroot”) AND (“male” OR “men” AND/OR “female” OR “women” OR “human”) AND (“sprint” OR “interval” OR “training” OR “performance” OR “ergogenic” OR “exercise” OR “short duration” OR “high intensity sprints”) AND (“power” OR “Wingate” OR “ergometer” OR “cycling”). The search results were downloaded into Zotero v.6. (Corporation for Digital Scholarship, USA) and imported into a systematic review-screening software (Covidence, AUS). Four authors (J.K.C., I.G.L., L.E.W., and M.J.N.) screened titles and abstracts to determine eligibility, removed duplicates, and any disagreements were resolved through consensus. Four authors (J.K.C., I.G.L., L.E.W., and M.J.N.) independently read and reviewed the articles and further eliminated articles based on the inclusion criteria. Additionally, four authors (J.K.C., I.G.L., L.E.W., and M.J.N.) conducted a reference analysis according to PICOS criteria (Table 1). 

The primary outcome variables included one or more of the following outcome variables: mean power output (P_mean_), peak power output (P_peak_), time-to-peak power, minimum power output (P_min_), and total work done, all measured on a cycle ergometer. Inclusion criteria were applied based on the Cochrane Risk of Bias 2 tool criteria.

### 2.2. Quality Assessment

Risk of bias was assessed with the Cochrane Risk of Bias tool for crossover trials (RoB 2) (Cochrane, Oxford, UK) by one author (M.J.N.) and verified by a second author (R.T.) [35]. The Cochrane Risk of Bias tool criteria included five components: (1) selection of the reported results, (2) measurement of the outcome, (3) missing outcome data, (4) deviations from intended interventions, and (5) randomization process. For each of the included studies, the Cochrane risk of bias tool for crossover trials criteria was categorized as “low risk”, “some concerns”, or “high risk”. Funnel plots and Egger’s regression tests were used to assess publication bias and were performed in Comprehensive Meta-Analysis version 4 software (Biostat Inc., Englewood, NJ, USA). In addition, quality assessment was also conducted using the Tool for the Assessment of Study Quality and Reporting in Exercise (TESTEX) scale by one author (J.K.C) and verified by a second author (T.J.M) [36]. We included both the Cochrane risk of bias tool and the TESTEX scale to account for study designs specific to exercise protocols.

### 2.3. Data Extraction

A standardized data extraction sheet was developed on Microsoft Excel to extract study characteristics and performance outcomes. Four authors independently extracted study details (J.K.C., I.G.L., L.E.W., and M.J.N.). A fifth author reviewed data extraction for accuracy and resolved any conflicts (R.T.). The data extracted included the following: sample size, participant characteristics (e.g., age, height, body mass, training status, and sex), supplementation regimen (dose, timing, frequency, and vehicle of administration), exercise protocol, exercise intensity, nitric oxide biomarker measurements and performance outcomes (P_mean_, P_peak_, time-to-peak power, P_min_, and total work done). One author (M.J.N.) completed data input of extracted data into the Comprehensive Meta-Analysis version 4 software (Biostat Inc., Englewood, NJ, USA) for statistical analysis. A second author verified the accuracy of the transferred data (R.T.). Mean and standard deviations were independently extracted by three authors (J.K.C., I.G.L., and L.E.W.) and reviewed by a fourth author (M.J.N.). 

### 2.4. Meta-Analyses 

Comprehensive Meta-Analysis version 4 software (Biostat Inc., Englewood, NJ, USA) was used for all analyses. Given the heterogeneity between studies (*a priori* significance was *p* < 0.05), a random-effects model was used to estimate the magnitude of effect of nitrate supplementation on performance variables. Hedges’ *g* effect sizes were calculated for each outcome; small, moderate, and large effects were defined as 0.20–0.49, 0.50–0.79, and ≥0.80, respectively [37]. Subgroup analyses were conducted for potential moderator variables of nitrate dose and dosing regimen. The pooled data for each primary outcome variable and subgroup analyses are presented as standardized mean differences (SMD), 95% confidence intervals (95% CI), and forest plots. If individual studies included multiple performance outcomes (e.g., P_peak_ in Wingate 1, Wingate 2, and Wingate 3), or a study was conducted in both sexes (e.g., Wingate 1 in men, Wingate 1 in women, etc.), SMDs were calculated for each of the performance variables measured within the study and were included in the same forest plot.

#### 2.4.1. Heterogeneity Assessment

Heterogeneity was assessed with Chi^2^ and *I*^2^ tests calculated in Comprehensive Meta-Analysis version 4 software (Biostat Inc., Englewood, NJ, USA) [38]. Values were defined as small (25–50%), medium (50–75%), and large (>75%) heterogeneity for *I*^2^, and significance was *p* ≤ 0.10 for Chi^2^ [38].

#### 2.4.2. Subgroup Analysis

Subgroup analyses were performed on: (1) nitrate dose (low: 5–8 mmol of nitrate i.e., 1 × 70 mL nitrate rich beetroot shots [Beet It; James Whyte Drinks, Ipswich, UK] vs. moderate: 11–13 mmol of nitrate i.e., 2 × 70 mL nitrate-rich beetroot shots [Beet It; James Whyte Drinks; UK]); (2) dosing regimen (acute vs. multiday); and (3) the number of cycling sprints completed. While a sex subgroup analysis was of interest, there was not enough representation of women to complete such an analysis (i.e., 10 women [39]; 2 women out of 13 participants [40]). 

## 3. Results

### 3.1. Study Selection

**Figure 1:**
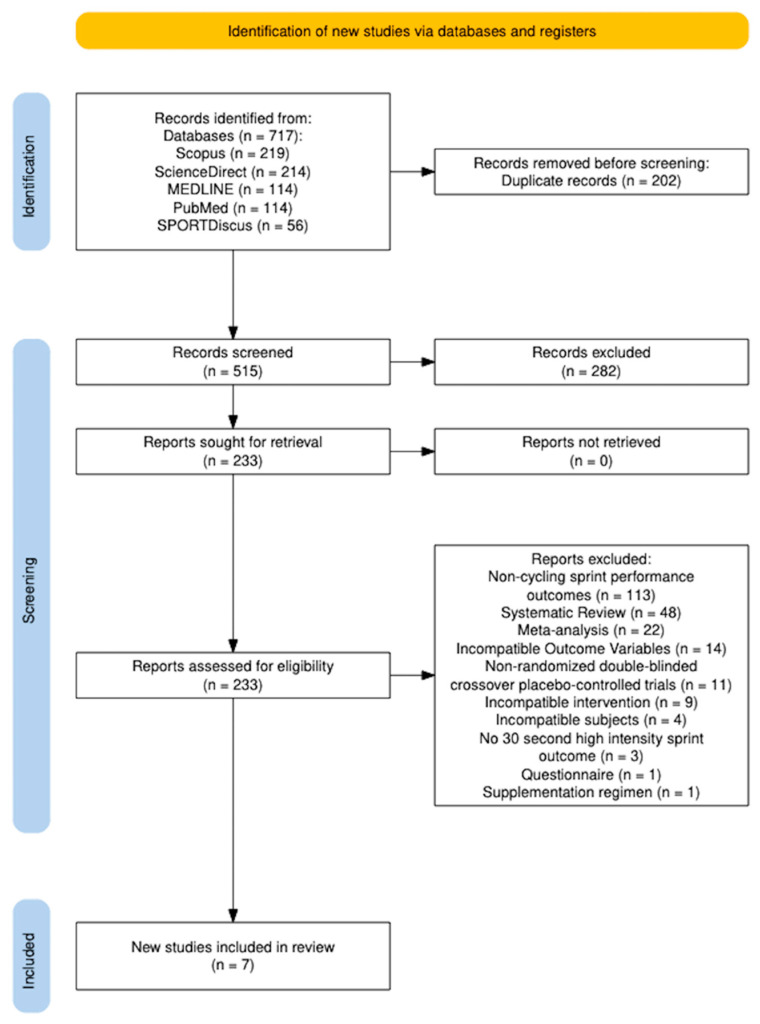
Literature search strategy.

The original search yielded a total of 717 results. After the elimination of duplicates, 233 full-text articles were eligible for review. A total of seven studies met the eligibility criteria for the present systematic review and meta-analysis (Figure 1). Absolute values for group mean and standard deviation data were not presented in table or text for three studies, and the authors were contacted [39,41,42]. Two authors responded and provided missing absolute values of mean and standard deviations [39,42]. Thus, we were unable to complete data extraction and data analysis for one study given that the absolute values of mean and standard deviations were not available in the full-text publication [41]. Means and standard deviations not provided in [39] were estimated from boxplots. Specifically, medians and first and third quartiles from Figure 3 in [39] were extracted using the PlotDigitizer app (https://plotdigitizer.com/; accessed on 5 August 2024) [43] and subsequently converted to means and standard deviations [44,45] (https://www.math.hkbu.edu.hk/~tongt/papers/median2mean.html; accessed on 5 August 2024). Finally, these means and standard deviations were combined (i.e., decomposed) into a single average and standard deviation (https://www.statstodo.com/CombineMeansSDs.php; accessed on 5 August 2024) for analysis. Therefore, a total of six studies were included for data extraction and data analysis.

### 3.2. Study Characteristics

A table summary of the six included studies is shown in Table 2. In the included studies, the sample size had a range of 10 to 20 participants, and the mean age range for the 88 total participants was 21 to 27 years. Participants were reported as having a range of training statuses using various standards, such as resistance-trained (i.e., three times per week for a minimum of 18 months and a bench press 1-repetition maximum 1 time higher than their body mass and a leg press 1-repetition maximum 1.5 times higher than their body mass [46] or three times per week in the past 18 months and a 1-repetition maximum greater than their body mass in bench press and 1.5 times greater than their body mass in back squats [42], experienced in Wingate tests (i.e., completed a Wingate at least once in the month before the visit to the lab) [47], recreationally active [39], athletes from various sports (tennis, alpine ski, American football, cycling, and triathlon) who were competitively trained [40], or recreational team-sport players familiar with intense intermittent exercise [48]. Participant data for calculating BMI were only available for three studies and resulted in a range of 23.7 to 24.2 kg·m^−2^ [39,42,47]. The supplementation methods administered were nitrate-rich beetroot juice (BR; Beet It; James Whyte Drinks; UK) containing 5.6 to ~13 mmol of nitrate in six studies [39,40,42,46,47,48]. Out of these six studies, for the placebo-control, only five studies administered a nitrate-depleted beetroot juice as the placebo control where the taste, smell, and appearance were identical to BR (PL; Beet It; James Whyte Drinks; UK) [39,40,42,46,48]; one study administered beetroot powder in mineral water mixed with lemon juice [47].

All studies performed exercise protocols on a cycle ergometer [39,40,42,46,47,48]. Four studies measured performance during a single 30-s “all-out” cycling test [40,42,46,47]. Two of these studies used the Wingate test model [42,47], one study conducted a maximal 30-s cycling trial after an inertial load cycling trial [40], and one study did not report the cycling load [46]. One study measured performance across three 30-s “all-out” non-Wingate tests (i.e., 30-s at 8.5% body weight) interspersed by 4 min of active recovery [39]. One study measured performance across seven repeated 30-s “all-out” non-Wingate tests interspersed with 4 min of recovery and did not report cycling load [48]. 

Five studies measured P_mean_ [39,42,46,47,48], six studies measured P_peak_ [39,40,42,46,47,48], four studies measured time-to-peak power [39,42,46,47], and three studies measured P_min_ [42,46,47]. Only one study measured total work done [40] and thus, this variable was not included in the analysis. Only one study included exclusive cohorts of women [39], while one study included 2 women out of 13 total participants [40]. Four studies included recreationally active adults [42,46,47,48]. One study included participants of different training statuses (recreationally active, competitive, elite) and reported metrics for training classifications, and given that there were no other studies with competitive or elite-level athletes, we only included data for the recreationally active participants in the analysis [39]. One study included participants from numerous university sports but did not report comprehensive training status classification (e.g., training load/history and/or maximal aerobic capacity), and therefore, these data were categorized as recreationally active participants [40]. 

Four studies provided a low nitrate dose (5.6 to 8.2 mmol of nitrate) [42,46,47,48], whilst two studies provided a moderate nitrate dose (11.2 to ~13 mmol of nitrate) [39,40]. Four studies administered acute nitrate supplementation 2.5 to 3 h prior to exercise [39,40,46,47]. Two studies administered multi-day nitrate supplementation protocols of five consecutive days of a moderate nitrate dose (8.2 mmol per day) [48] and six consecutive days of a moderate nitrate dose (~13 mmol per day) [39]. One study provided 11.2 mmol of nitrate across two doses that were administered 30 min apart, 2 to 3 h prior to exercise [40]. There were no studies that provided an elevated dose of nitrate (i.e., >13 mmol of nitrate).

Two studies measured plasma nitrate and nitrite via the gold standard of gas-phase chemiluminescence [39,48]. 

### 3.3. Quality Assessment

A summary of the quality assessment is presented in Supplementary Materials Figure S1, and an individual assessment of each study’s bias is presented in Supplementary Materials Figure S2. There were no studies excluded based on the Cochrane Risk of Bias tool scale or the TESTEX tool Supplementary Table S1.

Six studies had a low risk of bias in the overall bias domain [39,40,42,46,47,48]. Six studies had a low risk of bias in the selection of the reported result domain [39,40,42,46,47,48]. Five studies had a low risk of bias in the measurement of the outcome domain [39,40,42,46,47], and one study had a high risk of bias [48]. Six studies had a low risk of bias in the missing outcome data domain [39,40,42,46,47,48]. Five studies had a low risk of bias in the deviations from the intended interventions domain [39,40,42,46,47], and one study had some concerns [48]. Six studies had a low risk of bias in the bias arising from period and carryover effects domain [39,40,42,46,47,48]. Six studies had a low risk of bias in the randomization process domain [4,39,40,42,46,48]. 

### 3.4. Publication Bias

Although it is conventional to require 10 or more studies to reach adequate statistical power for funnel plots [49], these funnel plots were still calculated for all primary variables of interest. As none of the variables has 10 or more studies, all funnel plots were interpreted with caution and viewed as descriptive (as opposed to inferential) heuristics. Funnel plot figures are displayed in the Supplementary Materials (Figures S3–S6) for visual inspection of potential publication bias. 

Egger’s values are displayed in Supplementary Tables S2–S5. For P_mean_, the one- and two-tailed Egger’s *p*-values were 0.002 and 0.004, respectively. These results suggest the presence of publication bias for P_mean_, as both *p*-values were less than 0.10 [50]. For P_peak_, the one- and two-tailed Egger’s *p*-values were 0.04 and 0.07, respectively. These results suggest the presence of publication bias for P_peak_, as both *p*-values was less than 0.10 [50]. For time-to-peak power, the one- and two-tailed Egger’s *p*-values were 0.15 and 0.31, respectively. These results suggest that there was no publication bias for time-to-peak power, as both *p*-values were greater than 0.10 [50]. For P_min_, the one- and two-tailed Egger’s *p*-values were 0.08 and 0.16, respectively. The one-tailed (as opposed to the two-tailed) result suggested that publication bias may be a concern, as the *p*-value was less than 0.10 [50]. However, while as few as six studies may be appropriate for completing a publication bias analysis [50,51], caution is warranted, as less than 10 studies were included within this analysis [38].

### 3.5. Meta-Analysis

#### 3.5.1. Mean Power Output

Data for P_mean_ are displayed in the Supplementary Materials (Figure S7). Five studies measured P_mean_ [39,42,46,47,48]. There was no significant difference in P_mean_ following dietary nitrate supplementation (SMD: 0.16, 95% CI: −0.065 to 0.393, *p* = 0.16, *n* = 5). As the *Q*-value (1.82) was less than the degrees of freedom (6 − 1 = 5), the amount of between-study variance was less than expected (based on sampling error alone). As such, heterogeneity indices (e.g., *I*^2^) were estimated as zero.

#### 3.5.2. Peak Power Output

Data for P_peak_ are displayed in the Supplementary Materials (Figure S8). Six studies measured P_peak_ [39,40,42,46,47,48]. There was no significant difference in P_peak_ following dietary nitrate supplementation (SMD: 0.14, 95% CI: −0.073 to 0.349, *p* = 0.20, *n* = 6). As the *Q*-value (1.95) was less than the degrees of freedom (6 − 1 = 5), the amount of between-study variance was less than expected (based on sampling error alone). As such, heterogeneity indices (e.g., *I*^2^) were estimated as zero.

#### 3.5.3. Time-to-Peak Power 

Data for time-to-peak power are displayed in the Supplementary Materials (Figure S9). Four studies measured time-to-peak power [39,42,46,47]. There was a significance difference in time-to-peak power following dietary nitrate supplementation (SMD: −0.66, 95% CI: −1.127 to −0.192, *p* = 0.006, *n* = 4), and there was significant heterogeneity (Chi^2^ = 11.15; *I*^2^ = 64%; *p* = 0.025).

#### 3.5.4. Minimum Power Output

Data for P_min_ are displayed in the Supplementary Materials (Figure S10). Three studies measured P_min_ [42,46,47]. There was no significance difference in P_min_ following dietary nitrate supplementation (SMD: 0.12, 95% CI: −0.178 to 0.410, *p* = 0.44, *n* = 3). As the *Q*-value (0.69) was less than the degrees of freedom (3 − 1 = 2), the amount of between-study variance was less than expected (based on sampling error alone). As such, heterogeneity indices (e.g., *I*^2^) were estimated as zero.

#### 3.5.5. Subgroup Analyses: Mean Power Output

There were no statistical differences in subgroup analyses in for P_mean_ for dose, supplementation regimen, or number of sprints (Supplementary Materials Figures S11–S13).

#### 3.5.6. Subgroup Analyses: Peak Power Output

There were no statistical differences in subgroup analyses in for P_peak_ for dose, supplementation regimen, or number of sprints (Supplementary Materials Figures S14–S16).

#### 3.5.7. Subgroup Analyses: Time-to-Peak Power

Data for time-to-peak power subgroup analyses are displayed in the Supplementary Materials (Figures S17–S19). Time-to-peak power was significantly improved during one sprint following a low NO_3_^−^ dose provided acutely at 2–3 h prior to exercise (SMD: −0.977, 95% CI: −1.524 to −0.430, *p* < 0.001, *n* = 3) but not during three sprints when a moderate NO_3_^−^ dose was provided over 6 consecutive days (i.e., multiday) (SMD: −0.177, 95% CI: −0.619 to −0.264, *p* = 0.431, *n* = 2).

## 4. Discussion

The main finding of this systematic review and meta-analysis was that dietary nitrate supplementation enhanced time-to-peak power during single 30-s cycling sprints but had no effects on other power output metrics during single or multiple 30-s high-intensity cycling sprint exercise in recreationally active individuals. Notably, a limited number of studies were included, with variability in methodology and population, and thus, these findings should be treated with caution. In total, six studies were included, whereby five, six, four, and three studies measured P_mean_, P_peak_, time-to-peak power, and P_min_, respectively. Accordingly, these data should be treated as preliminary evidence that highlights the limited available data and serves as a call for researchers to investigate the efficacy of dietary nitrate supplementation on cycling sprint performance in various populations. 

To date, various meta-analyses have been conducted on the effects of dietary nitrate supplementation and exercise performance [20,52,53,54,55]. The overarching result from these meta-analyses is that dietary nitrate has a small-to-moderate effect size on time-to-exhaustion tests, while time trials were less likely to have any benefits [20,55]. Notably, the heterogeneity in methodology results in meta-analyses typically incorporating a myriad of exercise modalities (e.g., cycling, running, rowing, and kayaking [20,29] and/or exercise protocols (e.g., time trials, steady-state exercise, time-to-exhaustion trials, and sprints [53]). Thus, some of these results cannot be extrapolated to a specific exercise modality or exercise protocol, which precludes understanding whether nitrate can improve performance in a specific scenario such as cycling sprints. Our preliminary data highlight the need for further research on specific exercise protocols within specific exercise modalities to provide further insight as to whether dietary nitrate has ergogenic effects particularly on cycling sprints. 

The ergogenicity of dietary nitrate supplementation has been attributed to improved contractile function via enhanced type II muscle fiber calcium handling [21] and blood flow distribution [24]. Moreover, since type II muscle fiber recruitment is dependent on both the rate of force development and velocity of contraction [56,57], dietary nitrate supplementation has been suggested to potentially enhance high-power and high-velocity exercise performance [58] as well as peak power and contractile velocity [26]. Therefore, it is surprising that this meta-analysis found that dietary nitrate improved time-to-peak power but had no effect on mean power, peak power, or minimum power during 30-s Wingate and non-Wingate tests since these protocols require maximal “all-out” efforts that would be expected to recruit type II fibers [59]. However, these data may implicate that dietary nitrate ingestion may particularly benefit the initial phases of contraction, such as during acceleration phases, as suggested by previous studies [25,58,60], but should be interpreted with caution. Moreover, these results only apply to 30-s cycling sprints, but a plethora of intermittent-sprint protocols have been investigated (e.g., 6-s, 15-s, 20-s, 24-s, and 60-s cycling sprints [48,61,62,63]), which highlights the need for more research to understand how sprint protocols and supplementation regimen interact for enhancing performance. 

We found that an acute, low nitrate dose was effective at enhancing time-to-peak power, but a moderate nitrate dose ingested over multiple consecutive days was ineffective. While the reason for different performance effects between dosing regimens is currently unclear, it should be noted that numerous other factors may contribute to the efficacy of nitrate. Previous meta-analyses have demonstrated that factors such as supplementation protocol (e.g., timing, duration, frequency, and dosage), training status, exercise protocol, sex, and type of nitrate supplement likely impact the efficacy of dietary nitrate supplementation on exercise performance [20,64]. Notably, only one study included a sub-category cohort of recreationally active women (*n* = 10) and compared individuals across training statuses [39]. In addition, a limited number of studies have examined 30-s cycling protocols in elite-level athletes, which is a population that may be particularly interested in dietary nitrate supplementation [39]. Furthermore, further research is required to advance our understanding on how supplementation regimen (i.e., timing, dose, and acute vs. short-term vs. chronic supplementation) impacts the potential ergogenic effects of nitrate on cycling sprint performance. Together, more research is needed on women, varying training statuses, cycling sprints protocols, and supplementation regimen to increase the practical application of dietary nitrate to users who aim to enhance cycling sprint performance. For example, nitrate appears to have no effect on exercise performance in the majority of studies conducted in women thus far [65,66,67,68,69,70], and physiological sex-differences [71] may contribute to disparate results compared to men [20]. Therefore, this current paper serves as a call to researchers to continue investigating these factors. It should be noted that while the current study focused on power output metrics, we cannot exclude the possibility that nitrate could improve other aspects of cycling sprint performance, as demonstrated by others [29,48]. 

There are several limitations of this systematic review and meta-analysis. We included only six studies and double-counted one study in the analysis by including data from men and women within that study [39]. While this method has been previously employed in other meta-analyses [20,72], this might introduce bias towards this study, and thus, the results must be interpreted cautiously. Moreover, it is likely possible that the low number of studies, low sample sizes, and heterogeneity between the included studies contributed to the lack of ergogenic effect following nitrate supplementation, which highlights the need for further high-quality studies. Furthermore, only two studies included women [39,40], and only one study included elite-level athletes [39]. Therefore, we were unable to conduct subgroup analyses on sex-differences and training status. Lastly, only two studies included analytical procedures to measure nitric oxide bioavailability which verifies successful absorption and metabolism of dietary nitrate [39,48]. These data are critical, especially since performance has been associated with the magnitude of elevation in plasma [nitrite] (i.e., the surrogate biomarker for nitric oxide) [60,73], and future investigations are encouraged to include these analyses for robust procedures. 

## 5. Conclusions

The current systematic review and meta-analysis revealed that an acute, low nitrate dose improved time-to-peak power but not other power output metrics during 30-s high-intensity cycling sprints in recreationally active individuals. While these data represent a limited number of available studies and should be interpreted with caution, this meta-analysis may be an impetus for researchers to continue completing much-needed research to assess the potential of dietary nitrate supplementation to enhance sprint cycling performance.

## Figures and Tables

**Table 1:** PICOS criteria.

Parameter	Inclusion Criteria
Participant	Recreationally active, healthy adult males and females, aged 18–65 years.
Intervention	Nitrate supplementation provided as beetroot juice without contiguous ingestion of other supplements, and information was provided on the supplementation dose, timing, frequency, and vehicle of administration.
Comparator	Placebo provided as beetroot juice with negligible nitrate content.
Outcomes	Performance outcomes of peak power, mean power, time-to-peak power, minimum power, and total work done during bouts of 30-s high-intensity sprints.
Study Design	Randomized, double-blinded, crossover, placebo-controlled trials. Only studies that were published in English and as original research (i.e., not a conference abstract or review) were included.

**Table 2:** Studies assessing the effects of dietary NO_3_^−^ supplementation on cycling sprint performance in males.

Author	Subjects	Supplementation	Exercise Protocol	Findings
Cuenca et al. (2018) [46]	15 recreationally active men Age: 22.4 ± 1.6 y Ht: 1.78 ± 0.06 m Wt: 76.9 ± 10.3 kg	3 h prior to exercise ingestion of NO_3_^−^-rich BR (~6.4 mmol NO_3_^−^)	1 × CMJ PRE cycling sprint 30-s “all-out” cycling sprint 2 × CMJ POST cycling sprint	↑ P_peak_: +3.8% (PL: 848 ± 134 vs. BR: 881 ± 135) ↓ time-to-P_peak_: −18% (PL: 8.9 ± 1.4 vs. BR: 7.3 ± 0.9) ↑ P_mean_: + 4% (PL: 641 ± 91 vs. BR: 666 ± 100) ↔P_min_: 4.4% (PL: 8.9 ± 1.4 vs. BR: 7.3 ± 0.9)
Dominguez et al. (2017) [47]	15 recreationally active men Age: 21.46 ± 1.72 y Ht: 1.78 ± 0.07 m Wt: 76.90 ± 8.67 kg	3 h prior to exercise ingestion of NO_3_^−^-rich BR (~5.6 mmol NO_3_^−^)	30-s “all-out” cycling sprint at 7.5% body mass	↑ P_peak_: +5.4% (PL: 816.83 ± 136.97 vs. BR: 865.69 ± 143.91) ↔ P_mean_ (PL: 613.98 ± 94.14 vs. BR: 648.41 ± 104.79) ↓ time-to-P_peak_: −8.4% (PL: 8.00 ± 1.46 vs. BR: 7.33 ± 1.23) ↔ P_min_ (PL: 433.33 ± 99.39 vs. BR: 442.61 ± 122.79)
Jodra et al. (2019) [42]	15 recreationally active men Age: 23 ± 2 y Ht: 1.78 ± 0.06 m Wt: 75.6 ± 8.9 kg	3 h prior to exercise ingestion of NO_3_^−^-rich BR (~6.4 mmol NO_3_^−^)	1 × “all-out” cycling sprint at 7.5% body mass	↑ P_peak_: +4.4%: (PL: 848.40 ± 134.40 vs. BR: 880.93 ± 134.56) ↓ time-to-P_peak_ (PL: 8.87 ± 1.41 vs. BR: 7.33 ± 0.90) ↔ P_mean_ (PL: 641.14 ± 91.40 vs. BR: 666.48 ± 99.98) ↔ P_min_ (PL: 452.53 ± 64.63 vs. BR: 472.27 ± 72.35)
Jonvik et al. (2018) ** [39]	10 recreationally active men and 10 recreationally active women Age: men 27 ± 6 y women 33 ± 7 y Ht: men 1.84 ± 0.07 m women 1.70 ± 0.07 m Wt: men 78 ± 8 kg women 64 ± 8 kg	6 d NO_3_^−^-rich BR juice supplementation (~800 mg NO_3_^−^·d^−1^)	30-s “all-out” cycling sprint at 8.5% body mass	↓ time-to-P_peak_ (2.8%; *p* = 0.007) ↔ in P_mean_ (PL: 502.49 ± 56.40 vs. BR: 501.08 ± 53.99) (WG1 women) ↔ in P_peak_ (PL: 769.19 ± 108.25 vs. BR: 761.98 ± 105.64) (WG1 women) ↔ in P_mean_ (PL: 464.58 ± 54.89 vs. BR: 468.23 ± 44.06) (WG2 women) ↔ in P_peak_ (PL: 716.76 ± 102.16 vs. BR: 692.84 ± 97.02) (WG2 women) ↔ in P_mean_ (PL: 440.89 ± 54.88 vs. BR: 448.57 ± 49.56) (WG3 women) ↔ in P_peak_ (PL: 646.71 ± 89.40 vs. BR: 679.49 ± 97.42) (WG3 women) ↔ in P_mean_ (PL: 751.38 ± 69.56 vs. BR: 757.65 ± 59.40) (WG1 men) ↔ in P_peak_ (PL: 1260.24 ± 159.76 vs. BR: 1299.97 ±139.10) (WG1 men) ↔ in P_mean_ (PL: 676.16 ± 61.37 vs. BR: 671.91 ± 65.72) (WG2 men) ↔ in P_peak_ (PL: 1144.64 ± 175.18 vs. BR: 1157.27 ± 141.50) (WG2 men) ↔ in P_mean_ (PL: 632.65 ± 36.40 vs. BR: 614.18 ± 63.51) (WG3 men) ↔ in P_peak_ (PL: 1052.81 ± 149.37 vs. BR: 1050.79 ± 130.25) (WG3 men)
Rimer et al. (2016) [40]	11 men and 2 women university athletes Age: 25.9 ± 7.5 y Ht: 1.806 ± 0.075 m Wt: 73.8 ± 10.3 kg	2.5 h prior to exercise ingestion of NO_3_^−^-rich BR (~11.2 mmol NO_3_^−^)	4 × 3–4-s “all-out” cycling sprints + 120-s rest 1 × 30-s “all-out” cycling sprint (5 min after 4-s sprints)	↔ in P_peak_ (PL: 1185 ± 249 vs. BR: 1173 ± 255) ↔in total work (PL: 23.0 ± 4.4 KJ vs. BR: 22.8 ± 4.8 KJ)
Wylie et al. (2016) [48]	10 recreationally active men Age: 21 ± 1 y Ht: 1.82 ± 0.01 m Wt: 87.5 ± 9.5 kg	5 d NO^−^-rich BR juice supplementation (~8.2 mmol NO_3_^−^·d^−1^ + additional 4.1 mmol on day 3 and 4)	24 × 6-s “all-out” cycling sprints + 24-s passive recovery 7 × 30-s “all-out” cycling sprints + 210-s active recovery (20 W) + 30-s passive recovery 6 × 60-s cycling bouts (instructed to maximize mean power across all bouts) + 40-s active recovery (20 W) + 20-s passive recovery	7 × 30-s protocol: ↔P_mean_ (PL: 562 ± 94 vs. BR: 558 ± 95) ↔ P_peak_ (PL: 776 ± 142 vs. BR: 768 ± 157)

↑ = significant increase in BR vs. PL; ↓ = significant decrease in BR vs. PL; ↔ = no change; ** = average mean of all Wingate performance outcomes were estimated using an online calculator software; CMJ = counter movement jump; BR = beetroot juice; d = day; Ht = height; h = hours; kg = kilograms; m = meters; min = minutes; NO_3_^−^ = nitrate; PL = placebo; P_max_ = maximal power; P_mean_ = mean power; P_min_ = minimum power; P_peak_ = peak power; s = seconds; W = Watts; Wt: weight; WG1 = Wingate #1; WG2 = Wingate #2; WG3 = Wingate #3; y = years.

## Supplementary Materials

The following supporting information can be downloaded at: https://www.mdpi.com/article/10.3390/nu16162764/s1, Table S1: TESTEX Scale assessment of studies; Table S2: Egger’s regression intercept for P_mean_; Table S3: Egger’s regression intercept for P_peak_; Table S4: Egger’s regression intercept for time-to-peak power; Table S5: Egger’s regression intercept for P_min_. Figure S1: Summary risk of bias graph for double blinded randomized crossover trials evaluating the effects of dietary nitrate supplementation on performance outcomes during 30-s cycling sprints; Figure S2: Risk of bias for randomized double-blinded placebo-controlled trials; Figure S3: Funnel plot evaluating publication bias of trials assessing mean power output (P_mean_) following placebo and nitrate (n = 5). Jonvik et al. (2018) [39] was double-counted one time to account for data obtained in men and wome; Figure S4: Funnel plot evaluating publication bias of trials assessing peak power output (P_peak_) following placebo and nitrate (n = 6). Jonvik et al. (2018) [39] was double-counted one time to account for data obtained in men and women; Figure S5: Funnel plot evaluating publication bias of trials assessing time-to-peak power following placebo and nitrate (n = 4). Jonvik et al. (2018) [39] was double-counted one time to account for data obtained in men and women; Figure S6: Funnel plot evaluating publication bias of trials assessing minimum power (P_min_) following placebo and nitrate (n = 3); Figure S7: Forest plot demonstrating mean power output (P_mean_) following placebo (A) and nitrate (B); Figure S8: Forest plot demonstrating peak power output (P_peak_) receiving placebo (A) and nitrate (B); Figure S9: Forest plot demonstrating time-to-peak power following placebo (A) and nitrate (B); Figure S10: Forest plot demonstrating minimum power output (P_min_) following placebo (A) and nitrate (B). Figure S11: Forest plot demonstrating subgroup analysis by dose for following placebo (A) and nitrate (B) for P_mean_; Figure S12: Forest plot demonstrating subgroup analysis by supplementation regimen for following placebo (A) and nitrate (B) for P_mean_; Figure S13: Forest plot demonstrating subgroup analysis by number of sprints for following placebo (A) and nitrate (B) for P_mean_; Figure S14: Forest plot demonstrating subgroup analysis by dose for following placebo (A) and nitrate (B) for P_peak_; Figure S15: Forest plot demonstrating subgroup analysis by supplementation regimen for following placebo (A) and nitrate (B) for P_peak_; Figure S16: Forest plot demonstrating subgroup analysis by number of sprints for following placebo (A) and nitrate (B) for P_peak_; Figure S17: Forest plot demonstrating subgroup analysis by dose for following placebo (A) and nitrate (B) for time-to-peak power; Figure S18: Forest plot demonstrating subgroup analysis by supplementation regimen for following placebo (A) and nitrate (B) for time-to-peak power; Figure S19: Forest plot demonstrating subgroup analysis by number of sprints for following placebo (A) and nitrate (B) for time-to-peak power.
