# Supplementary material for: Effects of Dietary Nitrate Supplementation on High-Intensity Cycling Sprint Performance in Recreationally Active Adults: A Systematic Review and Meta-Analysis
Source: Nutrients. 2024 Aug 19;16(16):2764. doi: 10.3390/nu16162764 (PMC11357493; doi:10.3390/nu16162764)
Supplement: Supplementary file 1 [file nutrients-16-02764-s001.zip › nutrients-3140225-supplementary.pdf]

__Online Supplementary Material__

Search Strategy for Medline

S1 ("nitrate" OR "beetroot") AND ("male" OR "men" AND/OR "female" OR "women" OR "human") AND ("sprint" OR "interval" OR "training" OR "performance" OR "ergogenic" OR "exercise" OR "short duration" OR "high intensity sprints") AND ("power" OR "Wingate" OR "ergometer" OR "cycling") **(114)**

Search Strategy for PubMed

S2 ("nitrate" OR "beetroot") AND ("male" OR "men" AND/OR "female" OR "women" OR "human") AND ("sprint" OR "interval" OR "training" OR "performance" OR "ergogenic" OR "exercise" OR "short duration" OR "high intensity sprints") AND ("power" OR "Wingate" OR "ergometer" OR "cycling") **(114)**

Search Strategy for ScienceDirect

S3 ("nitrate" OR "beetroot") AND ("sprint" OR "ergogenic" OR "high intensity sprints") AND ("male" OR "female") AND ("power" OR "Wingate") **(214)**

Search Strategy for Scopus

S4 KEY(nitrate OR beetroot) AND (male OR men OR female OR women OR human) AND (sprint OR interval OR training OR performance OR ergogenic OR exercise OR short duration OR high intensity sprints) AND (power OR Wingate OR ergometer OR cycling) **(219)**

Search Strategy for SPORTDiscus

S5 ("nitrate" OR "beetroot") AND ("male" OR "men" AND/OR "female" OR "women" OR "human") AND ("sprint" OR "interval" OR "training" OR "performance" OR "ergogenic" OR "exercise" OR "short duration" OR "high intensity sprints") AND/OR ("power" OR "Wingate" OR "ergometer" OR "cycling") **(56)**

Table S1. TESTEX Scale assessment of studies.

Reference	Criterion 1	Criterion 2	Criterion 3	Criterion 4	Criterion 5	Criterion 6	Criterion 7	Criterion 8	Criterion 9	Criterion 10	Criterion 11	Criterion 12	Total
Cuenca et al. (2018)	1	1	1	1	1	2	1	2	1	1	1	1	14/15
Dominguez et al. (2017)	1	1	1	1	1	2	1	2	1	1	1	1	14/15
Jodra et al. (2019)	1	1	1	1	1	2	1	2	1	1	1	1	14/15
Jonvik et al. (2018)	1	1	1	1	1	2	1	2	1	1	1	1	14/15
Rimer et al. (2016)	1	1	1	1	1	2	1	2	1	1	1	1	14/15
Wylie et al. (2016)	1	1	1	1	1	2	1	2	1	1	1	1	14/15

Column criteria are according to TESTEX and are as follows (all criterion were worth 1 point unless denoted otherwise): Criterion 1= eligibility criteria specified; Criterion 2 = randomization specified; Criterion 3 = allocation concealment; Criterion 4 = groups similar at baseline; Criterion 5 = blinding of assessor; Criterion 6 (3 points) = outcome measurement assessed in 85% of patients (consists of three points: at least 85% adherence, adverse events, session attendance); Criterion 7 = intention to treat; Criterion 8 (2 points) = between group statistical comparisons (consists of two points; primary outcomes, and secondary outcomes); Criterion 9 = point measures; Criterion 10 = activity monitoring; Criterion 11 = relative exercise; Criterion 12 = exercise volume.

Table S2. Egger's regression intercept for P_{mean}.

Intercept	-5.74
Standard Error	0.97
95% lower limit (2-tailed)	-8.43
95% upper limit (2-tailed)	-3.05
t-value	5.93
Degrees of freedom	4.00
P-value (1-tailed)	0.002
P-value (2-tailed)	0.004

Table S3. Egger's regression intercept for P_{peak}.

Intercept	-4.39
Standard Error	1.95
95% lower limit (2-tailed)	-9.40
95% upper limit (2-tailed)	0.61
t-value	2.26
Degrees of freedom	5.00
P-value (1-tailed)	0.04
P-value (2-tailed)	0.07

Table S4. Egger's regression intercept for time-to-peak power.

Intercept	-9.54
Standard Error	7.77
95% lower limit (2-tailed)	-34.28
95% upper limit (2-tailed)	15.20
t-value	1.23
Degrees of freedom	3.00
P-value (1-tailed)	0.15
P-value (2-tailed)	0.31

Table S5. Egger's regression intercept for P_{min}.

Intercept	51.26
Standard Error	13.11
95% lower limit (2-tailed)	-115.26
95% upper limit (2-tailed)	217.77
t-value	3.91
Degrees of freedom	1.00
P-value (1-tailed)	0.08
P-value (2-tailed)	0.16

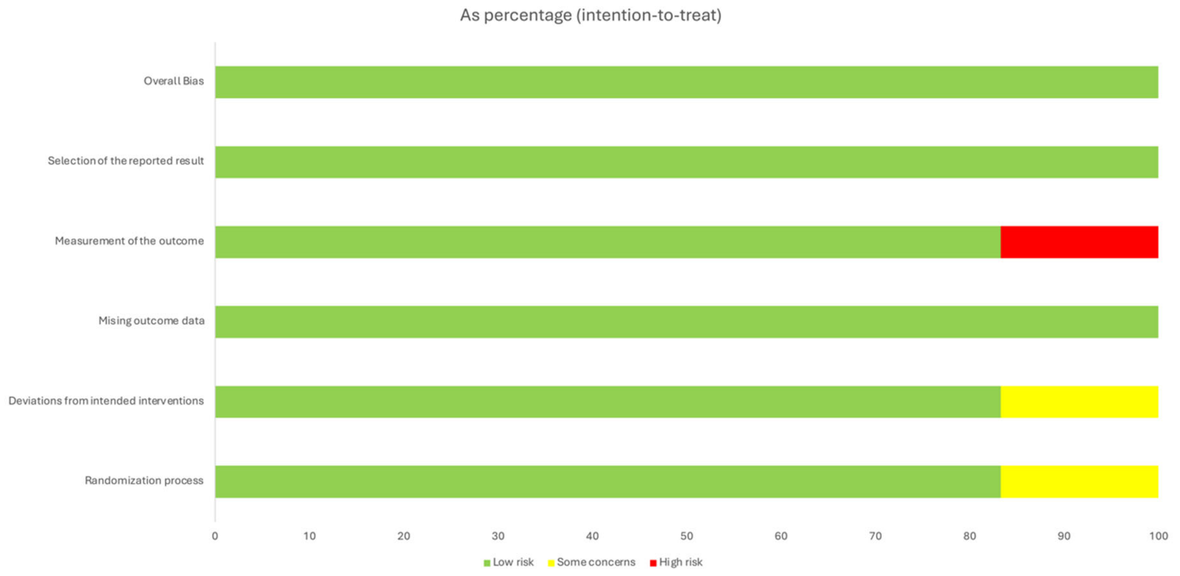

Figure S1. Summary risk of bias graph for double blinded randomized crossover trials evaluating the effects of dietary nitrate supplementation on performance outcomes during 30-second cycling sprints.

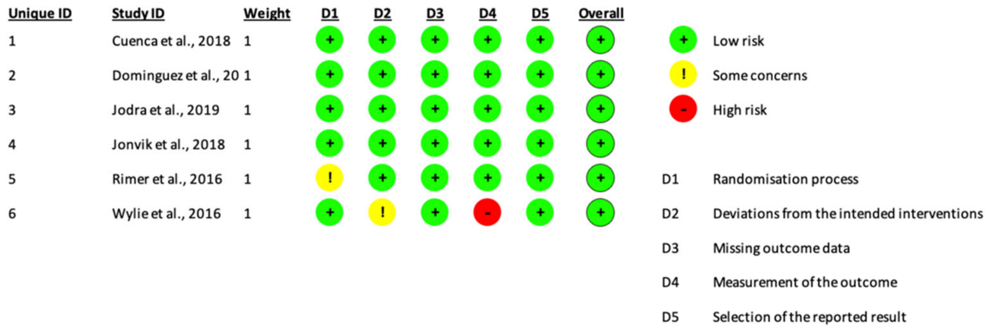

Figure S2. Risk of bias for randomized double-blinded placebo-controlled trials.

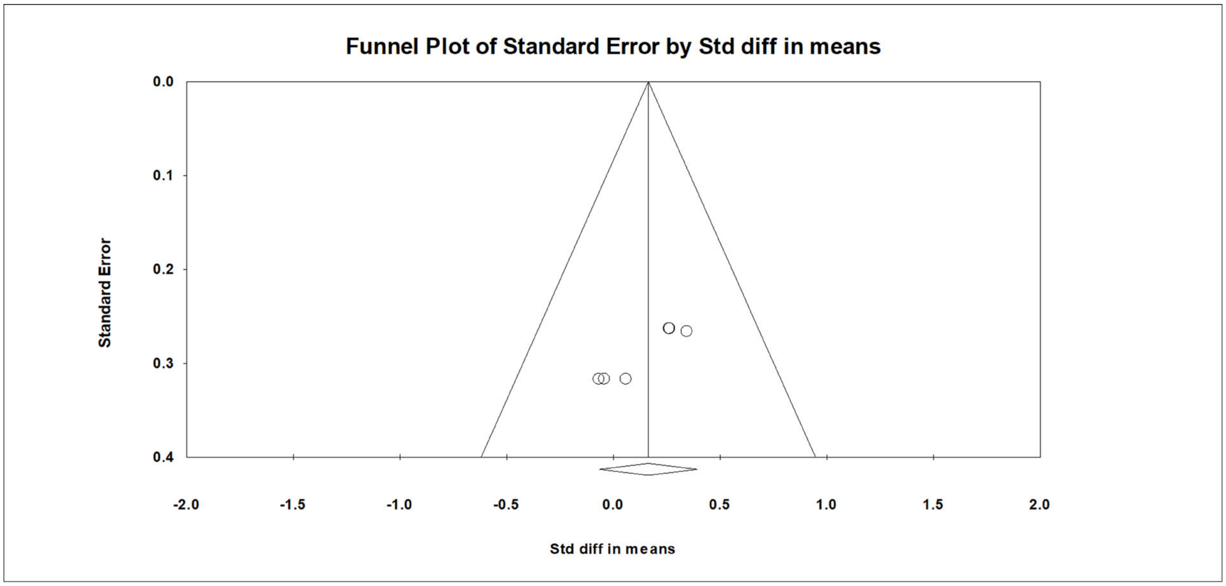

Figure S3. Funnel plot evaluating publication bias of trials assessing mean power output (P_{mean}) following placebo and nitrate (n=5). Jonvik et al. (2018) was double-counted one time to account for data obtained in men and women.

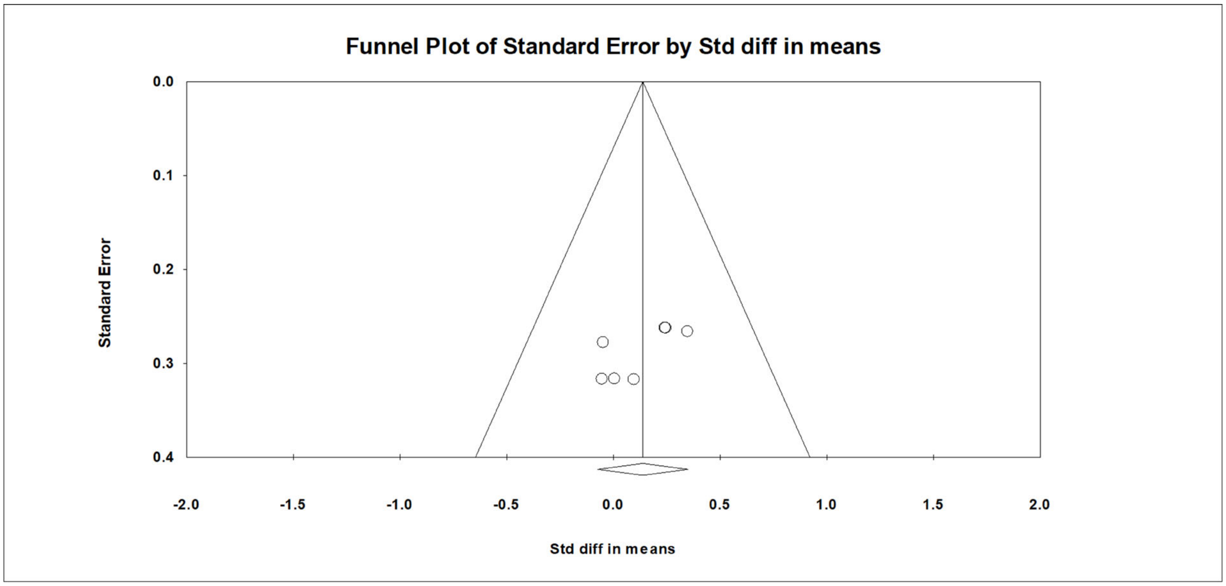

Figure S4. Funnel plot evaluating publication bias of trials assessing peak power output (P_{peak}) following placebo and nitrate (n=6). Jonvik et al. (2018) was double-counted one time to account for data obtained in men and women.

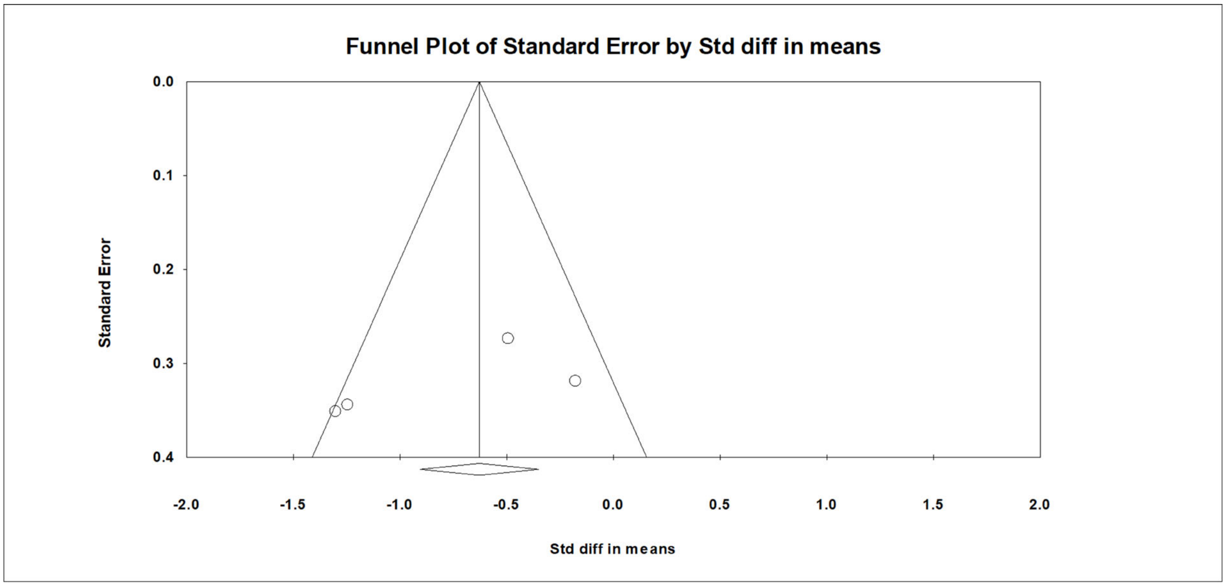

Figure S5. Funnel plot evaluating publication bias of trials assessing time-to-peak power following placebo and nitrate (*n*=4). Jonvik et al. (2018) was double-counted one time to account for data obtained in men and women.

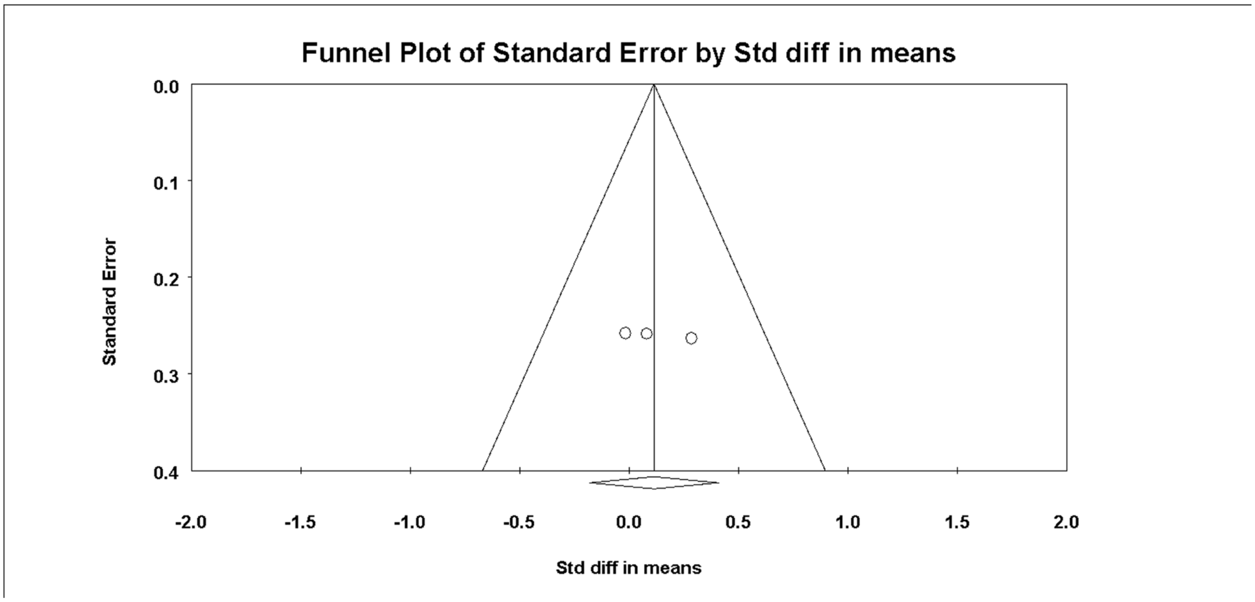

Figure S6. Funnel plot evaluating publication bias of trials assessing minimum power (P_{min}) following placebo and nitrate (n=3).

Study name	Subgroup within study	Std diff in means	Standard error	Variance	Lower limit	Upper limit	Z-Value	p-Value
		Statistics for each study						
Cuenca et al. (2018)	a	0.261	0.263	0.069	-0.254	0.776	0.994	0.320
Dominguez et al. (2017)	a	0.345	0.266	0.071	-0.176	0.866	1.297	0.195
Jodra et al. (2019)	a	0.264	0.263	0.069	-0.251	0.779	1.005	0.315
Jonvik et al. (2018)	g	-0.068	0.317	0.100	-0.689	0.552	-0.215	0.830
Jonvik et al. (2018)	h	0.059	0.317	0.100	-0.561	0.679	0.186	0.852
Wylie et al. (2016)	g	-0.042	0.316	0.100	-0.662	0.578	-0.134	0.894
Pooled		0.164	0.117	0.014	-0.065	0.393	1.401	0.161
Prediction Interval								

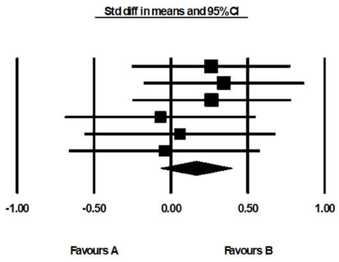

Meta Analysis

Figure S7. Forest plot demonstrating mean power output (P_{mean}) following placebo (A) and nitrate (B).

Study name	Subgroup within study	Std diff in means	Standard error	Variance	Lower limit	Upper limit	Z-Value	p-Value
Cuenca et al. (2018)	a	0.245	0.262	0.069	-0.268	0.759	0.936	0.349
Dominguez et al. (2017)	a	0.348	0.266	0.071	-0.174	0.869	1.307	0.191
Jodra et al. (2020)	a	0.242	0.262	0.069	-0.271	0.755	0.924	0.356
Jonvik et al.(2018)	g	0.097	0.317	0.100	-0.525	0.718	0.305	0.760
Jonvik et al.(2018)	h	0.005	0.316	0.100	-0.615	0.625	0.016	0.987
Rimer et al. (2016)	a	-0.048	0.278	0.077	-0.592	0.496	-0.172	0.864
Wylie et al. (2016)	g	-0.053	0.316	0.100	-0.674	0.567	-0.168	0.866
Pooled		0.138	0.108	0.012	-0.073	0.349	1.279	0.201
Prediction Interval								

Statistics for each study

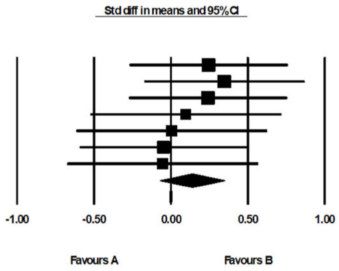

Meta Analysis

Figure S8. Forest plot demonstrating peak power output (P_{peak}) receiving placebo (A) and nitrate (B).

Study name	Subgroup within study	Std diff in means	Standard error	Variance	Lower limit	Upper limit	Z-Value	p-Value
Cuenca et al. (2018)	a	-1.302	0.351	0.123	-1.990	-0.614	-3.710	0.000
Dominguez et al. (2017)	a	-0.493	0.273	0.075	-1.029	0.043	-1.802	0.072
Jodra et al. (2019)	a	-1.245	0.344	0.118	-1.920	-0.571	-3.620	0.000
Jonvik et al. (2018)	g	-0.177	0.319	0.102	-0.802	0.447	-0.557	0.578
Jonvik et al. (2018)	h	-0.177	0.319	0.102	-0.802	0.447	-0.557	0.578
Pooled		-0.660	0.239	0.057	-1.127	-0.192	-2.766	0.006
Prediction Interval		-0.660			-2.214	0.895		

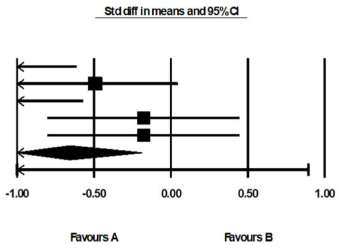

Meta Analysis

Figure S9. Forest plot demonstrating time-to-peak power following placebo (A) and nitrate (B).

Study name	Subgroup within study	Std diff in means	Standard error	Variance	Lower limit	Upper limit	Z-Value	p-Value
Cuenca et al. (2018)	a	-0.015	0.258	0.067	-0.521	0.491	-0.057	0.955
Dominguez et al. (2017)	a	0.082	0.259	0.067	-0.425	0.589	0.318	0.751
Jodra et al. (2020)	a	0.287	0.263	0.069	-0.230	0.803	1.089	0.276
Pooled		0.116	0.150	0.023	-0.178	0.410	0.772	0.440
Prediction Interval								

Statistics for each study

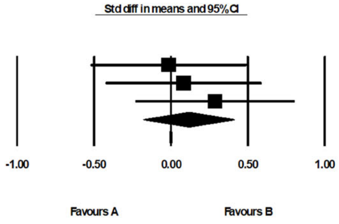

Meta Analysis

Figure S10. Forest plot demonstrating minimum power output (P_{min}) following placebo (A) and nitrate (B).

Group by Dose (mmol)	Study name	Subgroup within study	Std diff in means	Standard error	Variance	Lower limit	Upper limit	Z-Value	p-Value
Low	Cuenca et al. (2018)	a	0.261	0.263	0.069	-0.254	0.776	0.994	0.320
Low	Dominguez et al. (2017)	a	0.345	0.266	0.071	-0.176	0.866	1.297	0.195
Low	Jodra et al. (2019)	a	0.264	0.263	0.069	-0.251	0.779	1.005	0.315
Low	Pooled		0.289	0.152	0.023	-0.009	0.588	1.901	0.057
Low	Prediction Interval								
Moderate	Jonvik et al. (2018)	g	-0.068	0.317	0.100	-0.689	0.552	-0.215	0.830
Moderate	Jonvik et al. (2018)	h	0.059	0.317	0.100	-0.561	0.679	0.186	0.852
Moderate	Wylie et al. (2016)	g	-0.042	0.316	0.100	-0.662	0.578	-0.134	0.894
Moderate	Pooled		-0.017	0.183	0.033	-0.375	0.341	-0.094	0.925
Moderate	Prediction Interval								
Overall	Pooled		0.164	0.117	0.014	-0.065	0.393	1.401	0.161
Overall	Prediction Interval								

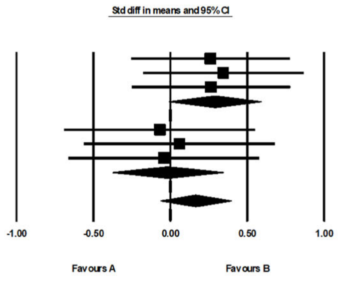

Meta Analysis

Figure S11. Forest plot demonstrating subgroup analysis by dose for following placebo (A) and nitrate (B) for P_{mean}.

Group by Regimen	Study name	Subgroup within study	Std diff in means	Standard error	Variance	Lower limit	Upper limit	Z-Value	p-Value
Acute	Cuenca et al. (2018)	a	0.261	0.263	0.069	-0.254	0.776	0.994	0.320
Acute	Dominguez et al. (2017)	a	0.345	0.266	0.071	-0.176	0.866	1.297	0.195
Acute	Jodra et al. (2019)	a	0.264	0.263	0.069	-0.251	0.779	1.005	0.315
Acute	Pooled		0.289	0.152	0.023	-0.009	0.588	1.901	0.057
Acute	Prediction Interval								
Multiday	Jonvik et al. (2018)	g	-0.068	0.317	0.100	-0.689	0.552	-0.215	0.830
Multiday	Jonvik et al. (2018)	h	0.059	0.317	0.100	-0.561	0.679	0.186	0.852
Multiday	Wylie et al. (2016)	g	-0.042	0.316	0.100	-0.662	0.578	-0.134	0.894
Multiday	Pooled		-0.017	0.183	0.033	-0.375	0.341	-0.094	0.925
Multiday	Prediction Interval								
Overall	Pooled		0.164	0.117	0.014	-0.065	0.393	1.401	0.161
Overall	Prediction Interval								

Statistics for each study

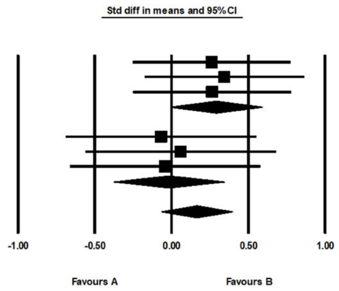

Meta Analysis

Figure S12. Forest plot demonstrating subgroup analysis by supplementation regimen for following placebo (A) and nitrate (B) for P_{mean}

Group by Number of sprints	Study name	Subgroup within study	Std diff in means	Standard error	Variance	Lower limit	Upper limit	Z-Value	p-Value
1.00	Cuenca et al. (2018)	a	0.261	0.263	0.069	-0.254	0.776	0.994	0.320
1.00	Dominguez et al. (2017)	a	0.345	0.266	0.071	-0.176	0.866	1.297	0.195
1.00	Jodra et al. (2019)	a	0.264	0.263	0.069	-0.251	0.779	1.005	0.315
1.00	Pooled		0.289	0.152	0.023	-0.009	0.588	1.901	0.057
1.00	Prediction Interval								
3.00	Jonvik et al. (2018)	g	-0.068	0.317	0.100	-0.689	0.552	-0.215	0.830
3.00	Jonvik et al. (2018)	h	0.059	0.317	0.100	-0.561	0.679	0.186	0.852
3.00	Pooled		-0.005	0.224	0.050	-0.443	0.434	-0.020	0.984
3.00	Prediction Interval								
7.00	Wylie et al. (2016)	g	-0.042	0.316	0.100	-0.662	0.578	-0.134	0.894
7.00	Pooled		-0.042	0.316	0.100	-0.662	0.578	-0.134	0.894
7.00	Prediction Interval								
Overall	Pooled		0.164	0.117	0.014	-0.065	0.393	1.401	0.161
Overall	Prediction Interval								

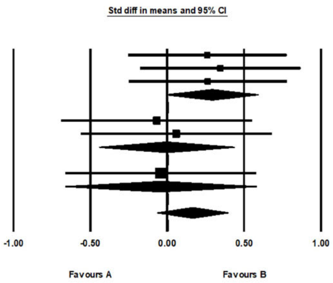

Meta Analysis

Figure S13. Forest plot demonstrating subgroup analysis by number of sprints for following placebo (A) and nitrate (B) for P_{mean}.

Group by Dose	Study name	Subgroup within study	Std diff in means	Standard error	Variance	Lower limit	Upper limit	Z-Value	p-Value
			Statistics for each study						
Low	Quenca et al. (2018)	a	0.245	0.262	0.069	-0.268	0.759	0.936	0.349
Low	Dominguez et al. (2017)	a	0.348	0.266	0.071	-0.174	0.869	1.307	0.191
Low	Jodra et al. (2020)	a	0.242	0.262	0.069	-0.271	0.755	0.924	0.356
Low	Wylie et al. (2016)	g	-0.053	0.316	0.100	-0.674	0.567	-0.168	0.866
Low	Pooled		0.216	0.137	0.019	-0.053	0.484	1.573	0.116
Low	Prediction Interval								
Moderate	Jonvik et al.(2018)	g	0.097	0.317	0.100	-0.525	0.718	0.305	0.760
Moderate	Jonvik et al.(2018)	h	0.005	0.316	0.100	-0.615	0.625	0.016	0.987
Moderate	Rimer et al. (2016)	a	-0.048	0.278	0.077	-0.592	0.496	-0.172	0.864
Moderate	Pooled		0.012	0.174	0.030	-0.329	0.354	0.069	0.945
Moderate	Prediction Interval								
Overall	Pooled		0.138	0.108	0.012	-0.073	0.349	1.279	0.201
Overall	Prediction Interval								

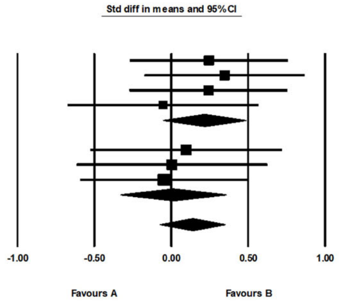

Meta Analysis

Figure S14. Forest plot demonstrating subgroup analysis by dose for following placebo (A) and nitrate (B) for P_{peak}.

Group by Regimen	Study name	Subgroup within study	Std diff in means	Standard error	Variance	Lower limit	Upper limit	Z-Value	p-Value
			Statistics for each study						
Acute	Cuenca et al. (2018)	a	0.245	0.262	0.069	-0.268	0.759	0.936	0.349
Acute	Dominguez et al. (2017)	a	0.348	0.266	0.071	-0.174	0.869	1.307	0.191
Acute	Jodra et al. (2020)	a	0.242	0.262	0.069	-0.271	0.755	0.924	0.356
Acute	Rimer et al. (2016)	a	-0.048	0.278	0.077	-0.592	0.496	-0.172	0.864
Acute	Wylie et al. (2016)	g	-0.053	0.316	0.100	-0.674	0.567	-0.168	0.866
Acute	Pooled		0.164	0.123	0.015	-0.077	0.405	1.335	0.182
Acute	Prediction Interval								
Multiday	Jonvik et al.(2018)	g	0.097	0.317	0.100	-0.525	0.718	0.305	0.760
Multiday	Jonvik et al.(2018)	h	0.005	0.316	0.100	-0.615	0.625	0.016	0.987
Multiday	Pooled		0.051	0.224	0.050	-0.388	0.490	0.227	0.820
Multiday	Prediction Interval								
Overall	Pooled		0.138	0.108	0.012	-0.073	0.349	1.279	0.201
Overall	Prediction Interval								

Std diff in means and 95%CI

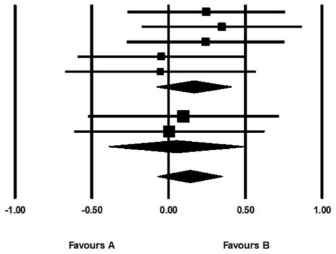

Meta Analysis

Figure S15. Forest plot demonstrating subgroup analysis by supplementation regimen for following placebo (A) and nitrate (B) for P_{peak}.

Group by Number of sprints	Study name	Subgroup within study	Std diff in means	Standard error	Variance	Lower limit	Upper limit	Z-Value	p-Value
1.00	Cuenca et al. (2018)	a	0.245	0.262	0.069	-0.268	0.759	0.936	0.349
1.00	Dominguez et al. (2017)	a	0.348	0.266	0.071	-0.174	0.869	1.307	0.191
1.00	Jodra et al. (2020)	a	0.242	0.262	0.069	-0.271	0.755	0.924	0.356
1.00	Rimer et al. (2016)	a	-0.048	0.278	0.077	-0.592	0.496	-0.172	0.864
1.00	Pooled		0.203	0.133	0.018	-0.059	0.464	1.519	0.129
1.00	Prediction Interval								
3.00	Jonvik et al (2018)	g	0.097	0.317	0.100	-0.525	0.718	0.305	0.760
3.00	Jonvik et al (2018)	h	0.005	0.316	0.100	-0.615	0.625	0.016	0.987
3.00	Pooled		0.051	0.224	0.050	-0.388	0.490	0.227	0.820
3.00	Prediction Interval								
7.00	Wylie et al. (2016)	g	-0.053	0.316	0.100	-0.674	0.567	-0.168	0.866
7.00	Pooled		-0.053	0.316	0.100	-0.674	0.567	-0.168	0.866
7.00	Prediction Interval								
Overall	Pooled		0.138	0.108	0.012	-0.073	0.349	1.279	0.201
Overall	Prediction Interval								

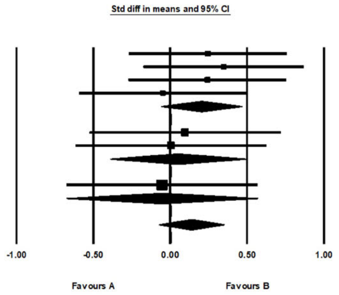

Meta Analysis

Figure S16. Forest plot demonstrating subgroup analysis by number of sprints for following placebo (A) and nitrate (B) for P_{peak}.

Group by Dose (mmol)	Study name	Subgroup within study	Std diff in means	Standard error	Variance	Lower limit	Upper limit	Z-Value	p-Value
Low	Cuenca et al. (2018)	a	-1.302	0.351	0.123	-1.990	-0.614	-3.710	0.000
Low	Dominguez et al. (2017)	a	-0.493	0.273	0.075	-1.029	0.043	-1.802	0.072
Low	Jodra et al. (2019)	a	-1.245	0.344	0.118	-1.920	-0.571	-3.620	0.000
Low	Pooled		-0.977	0.279	0.078	-1.524	-0.430	-3.503	0.000
Low	Prediction Interval		-0.977			-6.769	4.815		
Moderate	Jonvik et al. (2018)	g	-0.177	0.319	0.102	-0.802	0.447	-0.557	0.578
Moderate	Jonvik et al. (2018)	h	-0.177	0.319	0.102	-0.802	0.447	-0.557	0.578
Moderate	Pooled		-0.177	0.225	0.051	-0.619	0.264	-0.787	0.431
Moderate	Prediction Interval								
Overall	Pooled		-0.493	0.175	0.031	-0.837	-0.150	-2.814	0.005
Overall	Prediction Interval		-0.493			-1.960	0.973		

Statistics for each study

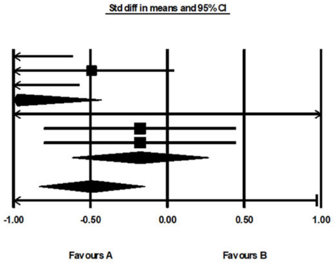

Meta Analysis

Figure S17. Forest plot demonstrating subgroup analysis by dose for following placebo (A) and nitrate (B) for time-to-peak power.

Group by Regimen	Study name	Subgroup within study	Std diff in means	Standard error	Variance	Lower limit	Upper limit	Z-Value	p-Value
			Statistics for each study						
Acute	Cuenca et al. (2018)	a	-1.302	0.351	0.123	-1.990	-0.614	-3.710	0.000
Acute	Dominguez et al. (2017)	a	-0.493	0.273	0.075	-1.029	0.043	-1.802	0.072
Acute	Jodra et al. (2019)	a	-1.245	0.344	0.118	-1.920	-0.571	-3.620	0.000
Acute	Pooled		-0.977	0.279	0.078	-1.524	-0.430	-3.503	0.000
Acute	Prediction Interval		-0.977			-6.769	4.815		
Multiday	Jonvik et al. (2018)	g	-0.177	0.319	0.102	-0.802	0.447	-0.557	0.578
Multiday	Jonvik et al. (2018)	h	-0.177	0.319	0.102	-0.802	0.447	-0.557	0.578
Multiday	Pooled		-0.177	0.225	0.051	-0.619	0.264	-0.787	0.431
Multiday	Prediction Interval								
Overall	Pooled		-0.493	0.175	0.031	-0.837	-0.150	-2.814	0.005
Overall	Prediction Interval		-0.493			-1.960	0.973		

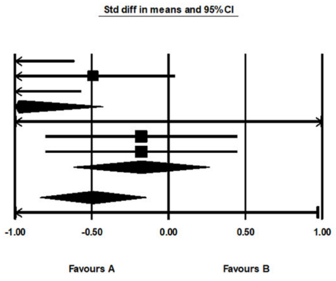

Meta Analysis

Figure S18. Forest plot demonstrating subgroup analysis by supplementation regimen for following placebo (A) and nitrate (B) for time-to-peak power.

Group by Number of sprints	Study name	Subgroup within study	Statistics for each study: Std diff in means	Standard error	Variance	Lower limit	Upper limit	Z-Value	p-Value
1.00	Cuenca et al. (2018)	a	-1.302	0.351	0.123	-1.990	-0.614	-3.710	0.000
1.00	Dominguez et al. (2017)	a	-0.493	0.273	0.075	-1.029	0.043	-1.802	0.072
1.00	Jodra et al. (2019)	a	-1.245	0.344	0.118	-1.920	-0.571	-3.620	0.000
1.00	Pooled		-0.977	0.279	0.078	-1.524	-0.430	-3.503	0.000
1.00	Prediction Interval		-0.977			-6.769	4.815		
3.00	Jonvik et al. (2018)	g	-0.177	0.319	0.102	-0.802	0.447	-0.557	0.578
3.00	Jonvik et al. (2018)	h	-0.177	0.319	0.102	-0.802	0.447	-0.557	0.578
3.00	Pooled		-0.177	0.225	0.051	-0.619	0.264	-0.787	0.431
3.00	Prediction Interval								
Overall	Pooled		-0.493	0.175	0.031	-0.837	-0.150	-2.814	0.005
Overall	Prediction Interval		-0.493			-1.960	0.973		

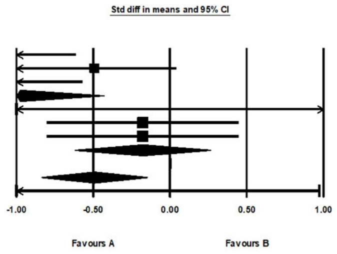

Meta Analysis

Figure S19. Forest plot demonstrating subgroup analysis by number of sprints for following placebo (A) and nitrate (B) for time-to-peak power.
